# Editorial: Updates on current protocols for the management of brain and spine malignancies

**DOI:** 10.3389/fneur.2022.1077973

**Published:** 2022-12-14

**Authors:** Tamara Ius, Luca Ricciardi, Giuseppe M. Barbagallo, Claudius Thomé, Antonino Raco

**Affiliations:** ^1^Division of Neurosurgery, AOU Sant'Andrea, Department of NESMOS, Sapienza University, Rome, Italy; ^2^Department of Medical and Surgical Sciences and Advanced Technologies (G.F. Ingrassia), University of Catania, Catania, Italy; ^3^Department of Neurological Surgery, Policlinico “G. Rodolico - San Marco” University Hospital, University of Catania, Catania, Italy; ^4^Interdisciplinary Research Center on Brain Tumors Diagnosis and Treatment, University of Catania, Catania, Italy; ^5^Department of Neurosurgery, Medical University Innsbruck, Innsbruck, Austria

**Keywords:** glioma, spinal tumor, brain, neuro-oncology, malignancy

Cerebral and spinal malignancies involve a highly complex anatomical region, in which a multidisciplinary approach is of utmost importance with regards to clinical management, surgical strategies, and adjuvant therapy. Local therapy with surgery and radiotherapy represents the most effective option both as first-line treatment and treatment at time of tumor recurrence. In recent years, the increasing knowledge in the molecular mechanisms of these tumors and translational research has allowed for new potential systemic treatments, which are constantly evolving. Despite growing knowledge of the molecular changes responsible for tumor development, glioblastoma remains a neoplasm with unmet medical needs, in which prognosis still remains poor. In addition, primary spinal malignancies still represent a challenging scenario for spine surgeons, in which surgical management is associated with high perioperative complication risk.

Our Editorial entitled “*Updates on current protocols for the management of brain and spine malignancies*” provides a general overview in this neuro-oncological setting, in terms of surgical strategy, adjuvant treatments, and molecular discoveries, emphasizing the fundamental role of a multidisciplinary approach tailored for each patient.

In the article entitled “*Pre- and post-surgical poor seizure control as hallmark of malignant progression in patients with glioma?*,” the emerging topic regarding the close relationship between epileptogenesis and oncogenesis is presented (Pauletto et al.). Pauletto et al. showed that a poor post-operative seizure outcome in LGG may correlate with a histological progression, highlighting the importance of a closer multidisciplinary follow-up for patients who are not seizure-free after surgery. The detection of early seizure recurrence in an important hallmark of malignant progression, especially in adult LGG patients.

With regards to high-grade gliomas (HGG), two interesting meta-analyses have been reported by Ricciardi, Sturiale et al.. In the study entitled “5*-Aminolevulinic acid false-positive rates in newly diagnosed and recurrent glioblastoma: Do pseudoprogression and radionecrosis play a role? A meta-analysis*,” 5-aminolevulinic acid (5-ALA) has been gradually used as a standard tool in neurosurgical procedures for HGG, providing a valuable increase in the extent of resection (EOR) (Ricciardi, Sturiale et al.). Its usefulness in terms of safety and accuracy for HGG recurrence has been reported in recent literature. In this precise systematic review and meta-analysis of comparative studies on the use of 5-ALA in newly diagnosed and recurrent GBM, the authors demonstrated that the 5-ALA plays a possible role in recurrent glioma surgery to appropriately guide surgical strategies. In the study entitled “*Carmustine Wafers implantation in patients with newly diagnosed high grade glioma: Is it still an option*?” the authors investigate the role of Carmustine Wafers in HGG (Ricciardi, Manini et al.). The results suggest that Carmustine Wafers implantation plays a significant role in improving survival when used in patients with newly diagnosed HGG. A careful patient selection is recommended (i.e., younger patients with a high probability of radical resection for small lesions) to minimize the risk of side effects.

Another area of interest which was included in our Research Topic was based on the role of surgery in elderly glioblastoma (GBM) patients. Approximately half of GBM cases occur in geriatric patients, and this trend is destined to increase with the aging of the population. In this clinical setting, Klingenschmid et al. analyzed 121 elderly GMB patients who underwent surgery. The authors reported that elderly patients who underwent a greater extent of resection of HGG lesions showed a significantly longer overall survival rate when compared to those patients that underwent biopsy or subtotal resection. The authors demonstrated that a good preoperative neurological status is a significant factor for overall survival, while the factor of age alone does not seem to influence the prognosis.

In newly diagnosed GBM patients, post-operative radiation with concurrent and adjuvant (six cycles) temozolomide (TMZ) is the standard of care. The potential benefit of extending adjuvant TMZ therapy beyond six cycles, however, remains questionable. To address this issue, Attarian et al.'s study compared the survival outcomes of standard TMZ and extended TMZ as the first-line treatment of patients with GBM. The authors concluded that extended TMZ beyond six cycles did not show an increase in progression-free survival or overall survival rate, thus addressing this important question in current neuro-oncological literature.

Despite the undisputed role of the Stupp protocol, GBM is considered an incurable disease, and the demand for new approaches and specific treatment options remains high. The targeted application of tumor-treating fields (TTFs) is a specific oncological option widely discussed and under investigation. The mechanism of action is based on the interference generated by the electrical fields on the mitotic activity of malignant cells. Krigers et al. evaluated a total of 48 patients harboring a GBM treated with TTF, demonstrating its efficacy in providing additional overall survival.

Novel targeted therapies are gradually changing the management and prognosis of HGG, especially at tumor recurrence. The identification of the oncogenic *FGFR3-TACC3* [*fibroblast growth factor receptor 3* (*FGFR3*)-*transforming acidic coiled-coil 3* (*TACC3*)] fusion highlighted the possibility of identifying a subset of diffuse glioma patients that seem to be potentially responsive to targeted therapy with *FGFR* kinase inhibitors. Broggi et al. describe an original case report and literature review on this emerging topic, emphasizing that an early identification of *FGFR3-TACC3* fusion may help select those patients that could potentially benefit from post-operative treatments with *FGFR* kinase inhibitors.

In the paper entitled “*Current and Future Frontiers of Molecularly Defined Oligodendrogliomas*,” Rincon-Torroella et al. summarized the current advancements in the molecular characterization of oligodendrogliomas. The optimal treatment paradigm for molecularly defined oligodendrogliomas is partially understood. The authors provided an extensive review regarding timing of radiation and chemotherapy, efficacy of different chemotherapeutic agents, and genetic factors influencing responsiveness to these agents.

With regards to spinal malignancies, progress has been made in the past years in the field of chemotherapy and radiation treatments. This has provided enhanced survival rates of oncological patients, which has also led to an increase of the number of patients with vertebral metastases. A multidisciplinary approach is of utmost importance, thus allowing for the integration of skills and knowledge of a team of specialists to ensure prompt diagnosis, support, and management of patients with spinal metastases and spinal cord compression. Rispoli et al. analyzed a large homogeneous cohort of 257 patients with vertebral metastasis. The study is based on an interesting multidisciplinary algorithm to optimize the outcomes of these patients. The authors underlined the importance of discussing each individual case during tumor board meetings in order to provide the best individualized, strategic options and management for each oncological patient.

Spinal metastases (SM) are one of the principal causes of morbidity and worsening of the quality of life (QoL) of oncological patients, mainly for neurologic involvement and intractable pain. Traditional open posterior instrumented fusion (OPIF) and percutaneous pedicle screw fixation (PPSF) represent the main surgical treatment alternatives for SM. There is no evidence in the current literature, however, that describes the absolute superiority of one treatment over the other. In recent years, the use of minimally invasive spinal surgery in SM patients has increased. Perna et al. conducted a systematic review and meta-analysis of comparative studies on PPSF vs. OPIF in patients with SM. The investigation pointed out that the PPSF treatment tends to lead to fewer complications, a lower rate of infections, a reduction in intraoperative blood loss, and a shorter hospital stay when compared to the OPIF treatment.

Unlike other areas of oncology, there are no current standardized markers or clinical indicators that can be used in the diagnosis, prognosis, and risk of recurrence in the field of neuro-oncology. Recent studies have shown the association between sarcopenia and mortality in SM. Tan et al. reported that sarcopenia could be a useful indicator of mortality in spinal metastasis patients. The authors suggested that this parameter could assist in the strategical decision-making process of treatment and management of these patients, even if the data is based on preliminary results and further long-term multicenter trials are needed.

Our Research Topic also touches on a very intriguing topic concerning the management of tumor rarities, such as primary central nervous system lymphomas (PCNSL), metastases of the hypothalamic–pituitary region, and primary sporadic intradural malignant peripheral nerve sheath tumor (MPNST). Scheichel et al. performed an interesting review on this topic, summarizing the diagnostic and surgery workup and carefully discussing the influence of preoperative corticosteroid therapy to reduce diagnostic delay in PCNLS patients. The authors underlined the importance of a multidisciplinary approach in PCNLS management, stressing the importance of timely therapy and providing a detailed systematic workflow for diagnosis.

Baiano et al. described the endoscopic endonasal approach for the management of hypothalamic–pituitary metastases. The authors detected recovery of visual field and improvement of oculomotor nerve palsy in 85.7 and 57.1% of cases, respectively, demonstrating that the endoscopic endonasal approach is a viable approach for the management of hypothalamic–pituitary metastases both in terms of neurovascular decompression and reliability in tissue sampling.

Primary sporadic intradural malignant peripheral nerve sheath tumor (MPNST) represents a rare and challenging disease, with an incidence of one case in 10 million. Spinal MPNSTs account for 2–3% of all MPNSTs, and primary sporadic intradural MPNSTs in the spinal canal are seen even less often. Cao et al. conducted an interesting systematic review, based on pooled data from 55 cases reported in the literature. The article expertly describes pathogenesis, clinical characteristics, imaging manifestations, differential diagnosis, surgical interventions, and pathological features in a systematic manner. The analysis demonstrated that even after surgical treatment and adjuvant treatment, the recurrence rate and mortality rate still tend to be high. Early detection and treatment are fundamental in MPNSTs management. The benefits of radiotherapy and chemotherapy treatments remain controversial, which thus underlies the importance of further multicenter studies.

As guest editors for this Research Topic, we hope you find the manuscripts prepared by our esteemed international colleagues innovative, practical, interesting, and of clinical value.

## Author contributions

All authors listed have made a substantial, direct, and intellectual contribution to the work and approved it for publication.

